# Phlorotannins and Fucans from Phaeophyceae as Phaeophyceae Agrochemical Leads: Structural Diversity, Molecular Mechanisms, and Delivery Systems

**DOI:** 10.3390/md24100340

**Published:** 2026-09-28

**Authors:** Isabelle Soares, Yasmim Leandro França do Nascimento, Evelize Folly das Chagas, Robson Xavier Faria, Valéria Laneuville Teixeira

**Affiliations:** 1 Postgraduate Program in Sciences and Biotechnology (PPBI), Fluminense Federal University, Niterói 24241-000, RJ, Brazil; soaaresisabelle@gmail.com (I.S.); yasmimfranca@id.uff.br (Y.L.F.d.N.); efolly@id.uff.br (E.F.d.C.); valerialaneuville@gmail.com (V.L.T.); 2 Laboratory for Environmental Health Assessment and Promotion (LAPSA), Oswaldo Cruz Institute, Rio de Janeiro 21040-360, RJ, Brazil; 3 Laboratory of Studies in Pest and Parasites (LEPP), Center for General Studies, Institute of Biology, Fluminense Federal University, Niterói 24210-200, RJ, Brazil; 4 Postgraduate of Neotropical Biodiversity, Rio de Janeiro 22290-255, RJ, Brazil

**Keywords:** brown algae, marine polyphenols, sulfated polysaccharides, secondary metabolites, biopesticides, crop protection, nanoencapsulation, blue-chemistry, sustainable agriculture

## Abstract

The increasing demand for sustainable agricultural practices has intensified the search for biobased alternatives to synthetic agrochemicals. In this context, marine macroalgae, particularly brown algae (Phaeophyceae), represent a largely underexplored reservoir of structurally diverse secondary metabolites with significant agrochemical potential. Among these, phlorotannins and sulfated polysaccharides such as fucoidans stand out because of their multifunctional biological activities. These compounds exhibit direct effects against phytopathogens and agricultural pests through mechanisms such as enzyme inhibition, disruption of cellular and midgut homeostasis, and interference with key metabolic pathways while also acting as elicitors of plant defense by activating systemic resistance responses. This review critically examines the structural diversity of phlorotannins and fucans from Phaeophyceae, correlating their molecular features with their reported biological activities and modes of action relevant to crop protection. Particular emphasis is placed on the chemical vulnerability of these metabolites under field conditions, where photodegradation, oxidative stress, and limited bioavailability restrict their practical application. Advances in green extraction technologies—such as ultrasound-assisted, microwave-assisted, and supercritical fluid extraction—are discussed as essential tools for preserving structural integrity and bioactivity. In parallel, state-of-the-art delivery systems, including nanoencapsulation using biopolymers such as alginate and chitosan, are highlighted as effective strategies to enhance stability, controlled release, and environmental persistence. Finally, the review addresses critical challenges related to the extraction of standardization, formulation scalability, and regulatory frameworks that currently limit the commercial adoption of marine-derived agrochemicals. By integrating metabolic diversity, green chemistry, and advanced formulation technologies, Phaeophyceae-derived metabolites have emerged as promising leads for the development of innovative, low-impact agrochemicals that are compatible with integrated best management strategies and the principles of blue chemistry.

## 1. Introduction

### 1.1. Bioactive Chemistry of Phaeophyceae and Prospects for Integrated Pest Management

Contemporary agriculture faces profound challenges associated with the accelerated evolution of resistance in arthropod and nematode populations—a phenomenon that has progressively undermined the long-term efficacy of synthetic agrochemicals. This selective pressure, coupled with increasingly stringent regulatory frameworks aimed at reducing the reliance on conventional pesticides, has intensified the search for environmentally compatible and sustainable alternatives, as illustrated in the Graphical Abstract [1].

Within this context, the bioprospecting of marine organisms has gained prominence as a strategic approach for the discovery of novel bioactive compounds. Among marine resources, the class Phaeophyceae (brown algae) stands out as a largely underexplored reservoir of secondary metabolites with significant phytosanitary potential. The biological success of these macroalgae is underpinned by a distinctive metabolic framework characterized by the production of specialized polysaccharides and polyphenolic compounds that are rarely encountered in terrestrial plants [1].

#### Phaeophyceae Metabolism: Structural Diversity and Biosynthesis

The agrochemical and pharmacological relevance of brown algae is intrinsically linked to their ability to biosynthesize a chemically diverse array of secondary metabolites. A detailed understanding of the structure–function relationships governing these compounds is essential for identifying effective lead molecules for pest management applications. The secondary metabolism of Phaeophyceae is characterized by remarkable structural diversity and includes terpenoids, polyphenols, sulfated and nonsulfated polysaccharides, and distinct oxylipins [2,3].

Brown algae produce a variety of bioactive secondary metabolites with structural features unique to marine environments. These compounds arise via complex biosynthetic pathways that confer multifunctional biological properties, positioning brown algae as a significant source of agrochemical and pharmacological leads. This agrochemical relevance is particularly compelling given that many of these compounds have evolved as integral components of marine defense strategies against herbivory, fouling, and environmental stress [4]. Key metabolite classes include phlorotannins—polyphenolic compounds with well-documented antioxidant, antifeedant, and herbicidal properties—as well as structurally diverse terpenoids and halogenated metabolites that exhibit antimicrobial and antifungal activities [5].

The biosynthesis of these molecules frequently involves enzymatic oxygenations, halogenations, and oxidative coupling reactions that introduce functional groups essential for biological activity. Core biosynthetic routes have been highly adapted to the marine environment; specifically, phlorotannins are synthesized through the polymerization of phloroglucinol units via the acetate–polymalonate pathway, a route distinct from the shikimic acid pathway typical of terrestrial plants. This metabolic adaptation results in chemical structures with optimized stability and reactivity tailored to oceanic ecosystems [6].

Notably, in brown algae, phlorotannins are biosynthesized predominantly—if not exclusively—via the acetate–malonate (polyketide) pathway, which is metabolically linked to terpenoid biosynthesis, rather than through the shikimate pathway that dominates polyphenol formation in terrestrial plants. Only specific classes of aromatic metabolites in Phaeophyceae appear to involve shikimate-derived intermediates, underscoring the unique evolutionary origin of algal polyphenols. Structural diversity directly influences the biological function of these metabolites by mediating interactions with biological targets, such as insect nervous systems or fungal enzymes [6].

These structure–activity relationships (SAR) are central to the development of effective agrochemical leads, as subtle variations in molecular architecture govern potency, selectivity, and environmental persistence [7]. For example, oxylipins produced enzymatically or nonenzymatically in brown algae are associated with innate immune responses and chemical defense signaling, highlighting their potential as natural pesticides or deterrents. Moreover, the selective incorporation of functional groups by Phaeophyceae-specific enzymes allows fine-tuning of hydrophobicity and binding affinity—two parameters that are critical determinants of agrochemical performance [8].

### 1.2. Bioactivity Potential of Marine Phaeophyceae

Marine macroalgae belonging to the class Phaeophyceae constitute an exceptional source of structurally unique secondary metabolites that differ substantially from those produced by terrestrial vascular plants. Among these compounds, phlorotannins and fucoidans are particularly notable for their broad spectrum of biological activities and considerable biotechnological relevance. Phlorotannins, a class of polyphenols exclusive to brown algae, are complex polymers derived from phloroglucinol units that play essential physiological roles, including chemical defense against herbivores, antioxidant protection, and structural reinforcement of the algal cell wall [1,9].

Extensive experimental evidence has identified these compounds as promising bioactive agents possessing antimicrobial, antifungal, antiviral, larvicidal, and antifeedant properties. Consequently, phlorotannins and related metabolites have emerged as attractive natural precursors for the development of novel biopesticides and sustainable alternatives to synthetic agrochemicals [9,10,11].

## 2. Methodology of Literature Review

This narrative review was conducted through a comprehensive search of electronic databases, including Web of Science, PubMed, Scopus and Google Scholar, encompassing publications from 2000 to 2026. The search strategy employed specific keywords and Boolean operators: (“brown algae” OR “Phaeophyceae”) AND (“phlorotannins” OR “fucans”) AND (“biopesticide” OR “agrochemical” OR “delivery systems”). The inclusion criteria focused on peer-reviewed articles, book chapters, and patents addressing structural diversity, molecular mechanisms, and industrial applications. Studies were selected on the basis of their alignment with the “structure–function–application–optimization” framework.

## 3. Chemical Diversity of Bioactive Compounds in Phaeophyceae

The chemical landscape of Phaeophyceae is characterized by a unique repertoire of secondary metabolites that distinguish these marine organisms from terrestrial flora. Among these compounds, phlorotannins constitute the most prominent class of phenolic compounds and function as essential components of both the cell wall matrix and intracellular vesicles. These compounds are complex oligomers or polymers synthesized exclusively through the polymerization of phloroglucinol (1,3,5-trihydroxybenzene) units, which serve as the fundamental monomeric building blocks [12,13,14].

The structural diversity of phlorotannins is vast, stemming from variations in molecular weight—reaching degrees of polymerization (DP) of up to 27 units—and the high degree of isomerism afforded by diverse branching patterns and oxidative coupling mechanisms [12,13,14].

### 3.1. Phlorotannins: Oligomers of Phloroglucinol

Phlorotannins represent a highly specialized class of phenolic oligomers found exclusively in brown algae and are synthesized via the oxidative polymerization of phloroglucinol units (1,3,5-trihydroxybenzene), as schematically represented in Figure 1. The remarkable diversity of these compounds is primarily dictated by the nature of the interunit linkages connecting the monomeric subunits.

Fucols are defined by direct aryl–aryl (C-C) bonds, enabling the formation of linear and branched polymeric chains through direct carbon–carbon coupling. In contrast, phlorethols and fuhalols are characterized by ether (C-O-C) linkages; fuhalols are distinguished from phlorethols by the presence of additional hydroxyl groups, which significantly increase their molecular complexity and chemical reactivity. Fuhalols, a more structurally rigid subclass, are characterized by the inclusion of at least one dibenzo-1,4-dioxin moiety. This tricyclic ring system originates from intramolecular oxidative coupling and confers enhanced conformational stability relative to that of other phlorotannin classes.

As depicted in complex polymer models, the linkage type and molecular architecture—especially those observed in cell-wall-bound phlorotannins from *Fucus vesiculosus*—are critical determinants of biological functionality and clearly distinguish these compounds from structurally simpler intracellular variants [1,15].

The biological efficacy of metabolites derived from Phaeophyceae is fundamentally governed by their intrinsic structural motifs and precise structure–activity relationships (SAR). A critical determinant of this activity is the density of hydroxyl (-OH) groups, which serve as essential coordination sites for metal chelation and the mitigation of environmental toxicity. Furthermore, an increased degree of polymerization (DP), coupled with a higher abundance of hydroxyl moieties, enhances the thermodynamic stability and buffering capacity of the resulting metal–ligand complexes. This mechanism not only underpins the physiological defense strategies of algae but also highlights their potential use in targeted environmental detoxification.

Beyond general bioactivity, these metabolites exhibit selective interactions with biochemical targets dictated by their specific molecular configurations. For instance, phlorotannins characterized by a high density of free hydroxyl groups function as potent competitive inhibitors of acetylcholinesterase (AChE) in pests such as *Spodoptera frugiperda*. This inhibition is mediated by the facilitation of ᴨ-ᴨ stacking interactions between the phenolic rings and the aromatic residues within the enzyme’s active site—a phenomenon notably more pronounced in complex eckol-type phlorotannins than in monomeric phloroglucinol units. Similarly, the degree of sulfation in fucans is a primary determinant of their binding affinity toward the cell membranes of phytopathogens. In *Sargassum* spp., highly sulfated fractions interact electrostatically with the positively charged residues of fungal membrane proteins, inducing cytoplasmic leakage and a concomitant reduction in minimum inhibitory concentration (MIC) values. Such structural complexity further dictates host–pathogen selectivity; for example, low-molecular-weight fucoidans preferentially inhibit cell wall-degrading enzymes (CWDEs) in *Fusarium* species. This refined SAR ensures targeted efficacy while maintaining the integrity of beneficial soil microbiota, thereby avoiding nontargeted toxicity [9].

Moreover, the density of hydroxyl groups strongly influences the toxicity profile of phlorotannins. In general, these compounds exhibit low cytotoxicity toward mammalian and plant cell lines—a property largely attributed to their high polarity and limited membrane permeability, which restricts their intracellular accumulation. Conversely, the antioxidant capacity of phlorotannins, mediated through free radical scavenging, is positively correlated with the number of hydroxyl substituents on the aromatic rings. Although lower-molecular-weight oligomers may display distinct bioavailability profiles, higher-molecular-weight polymers typically provide superior protection against oxidative stress [11,16].

Notably, SAR studies of phenolic compounds indicate that chemical modifications such as hydroxyl methylation may increase lipophilicity and potency; however, the naturally polyhydroxylated architecture of phlorotannins appears to favor an optimal balance between high bioactivity and systemic safety [11,16]. Consequently, the intricate organization of phloroglucinol units not only underpins diverse ecological roles but also establishes phlorotannins as robust molecular templates for sustainable agrochemical development.

The systematic classification of phlorotannins according to linkage chemistry provides a valuable framework for understanding their divergent biological properties and their suitability as agrochemical leads. As summarized in Table 1, these phenolic compounds are grouped into major classes defined by the oxidative coupling of pattern phloroglucinol subunits. Fucols exhibit direct carbon–carbon (aryl–aryl) linkages and are found predominantly in species such as *Fucus vesiculosus* and *Ascophyllum nodosum* (Fucales). Phlorethols and fuhalols characterized by ether (C-O-C) bonds differ in terms of their hydroxyl group density, with the polarity and reactivity of these compounds increasing. Eckols, identified mainly in *Ecklonia cava* and *Eisenia bicyclis*, incorporate a dibenzo-1,4-dioxin moiety that impacts pronounced structural rigidity, often correlating with enhanced enzymatic inhibitory activity. Mixed-linkage classes, such as ishigeols, found in *Ishige okamurae*, contain both aryl–aryl and ether bonds within a single molecular framework [12,13].

The structural complexity of phlorotannins is further amplified by their broad molecular weight distribution, which varies significantly across different species and environmental conditions. Advanced mass spectrometric analyses have revealed that the abundance of phlorotannins ranges from dimers to polymers exceeding 100 kDa; nevertheless, compounds with DPs between 3 and 27 are most frequently isolated and evaluated for their biological activity [12,13].

This molecular diversity is not merely a chemical curiosity but a defining attribute of the “blue chemistry” potential of Phaeophyceae. For example, the rigid frameworks of eckols often translate into enhanced stability and specific binding affinities in enzyme inhibition assays—critical parameters for the development of durable biopesticides. Furthermore, the high density of reactive hydroxyl groups across various phlorotannin classes promotes the formation of stable complexes with metallic ions and organic molecules, thereby improving environmental persistence and bioefficacy under field conditions. Exploiting these structure–activity relationships enables the rational stabilization of marine-derived leads and facilitates their translation into reliable agrochemical products [9,14].

#### 3.1.1. Structural Classification of Phlorotannins

The structural diversity of these compounds is directly linked to their biological efficacy. For instance, eckols such as dieckol and 6,6-bieckol have demonstrated potent inhibitory effects against agricultural pathogens, whereas fuhalols are known for their specific ether-linked patterns that influence their antioxidant and herbicidal potential. Fucols and ishigeols also exhibit distinct bioactivity profiles because of their unique aryl–aryl and ether connectivity, respectively. Phlorotannins constitute a unique class of polyphenolic compounds found exclusively in brown algae (Phaeophyceae). These secondary metabolites are biosynthesized via the acetate–polymalonate pathway as polymers of phloroglucinol (1,3,5-trihydroxybenzene). Their structural classification is primarily determined by the specific linkage patterns between phloroglucinol units and the degree of polymerization. On the basis of these interunit linkages, phlorotannins are categorized into several distinct subclasses:

- Fucols: Characterized by aryl–aryl (C-C) bonds, these molecules result from direct carbon–carbon coupling between aromatic rings, with difucol serving as a fundamental representative [15].
- Phlororethols (Florethlos): These polymers are connected via diaryl ether (C-O-C) bridges. This oxygen-bridged configuration introduces greater conformational flexibility and distinct chemical properties than fucols do [14,15].
- Fuhalols: While also containing ether linkages, fuhalols are distinguished by additional hydroxyl substitutions on the phloroglucinol units. This increased hydroxylation enhances their polarity and bioactivity, making them the dominant phlorotannins in species such as *Sargassum fusiforme* [15].
- Fucophlorethols: This subclass exhibits a hybrid structural arrangement consisting of both aryl–aryl (C-C) and diaryl ether (C-O-C) linkages. Such mixed-bond configurations have been identified in *Ascophyllum nodosum* and related species [14,15].
- Eckols: In addition to having at least one dibenzo-1,4-dioxin linkage, eckols possess a unique bicyclic structural motif. A prominent example is dieckol (C_30_H_18_O_14_). This dibenzodioxin marker is characteristic of eckol-type phlorotannins found in the cell walls of fucoid algae, including *Fucus vesiculosus* and *Pelvetia canaliculata* [1,14].
- Inshigeols: This specific class is associated with the genus *Ishige* and displays unique ether/aryl bonding patterns. Although less extensively characterized than other classes are, ishigeols represent a taxonomically significant group [15].

#### 3.1.2. Structural Diversity and Analytical Implications

The chemical complexity of phlorotannins is further amplified by significant isomer diversity. For a given molecular weight, numerous structural isomers can exist because of variations in linkage positions and branching patterns. For instance, research on *Fucus vesiculosus* has revealed up to 61 distinct isomers for a single degree of polymerization (approximately 12 units). Such structural heterogeneity poses substantial challenges for natural product characterization, necessitating the use of advanced analytical platforms, such as ultrahigh-performance liquid chromatography coupled with high-resolution tandem mass spectrometry (UHPLC-HRMS/MS), to accurately profile these bioactive compounds [13,15].

### 3.2. Fucans and Sulfated Polysaccharides

Fucoidans and related sulfated polysaccharides derived from Phaeophyceae constitute a structurally diverse group of macromolecules characterized by a complex backbone primarily composed of α-L-fucopyranosyl residues. These polymers frequently manifest as heterofucans or sulfated galactofucans, incorporating varying proportions of neutral sugars—such as galactose, glucose, and xylose—as well as uronic acids, all of which contribute to pronounced structural heterogeneity [21,22,23].

The biological activity of these polyanionic carbohydrates is fundamentally governed by both the degree and regioselectivity of sulfation. The precise distribution of sulfate ester groups, particularly at the C-2 or C-4 positions, dictates molecular recognition events and selective binding to cellular receptors and signaling proteins, extending beyond simple electrotactic interactions [24].

The rheological properties of these polysaccharides—modulated by molecular weight, solubility, and chain conformation—are closely linked to their interaction with pest cellular membranes. Defined sulfation patterns regulate associations with membrane-anchored proteins and phospholipids, potentially compromising membrane integrity and disrupting essential signal transduction pathways. Notably, fucose-rich sulfated polysaccharides can induce apoptosis in various cellular systems through alterations in membrane asymmetry and the activation of intracellular caspase cascades [23].

Furthermore, species-specific membrane interactions mediated by precise sulfation motifs, as documented in marine biological models, highlight the feasibility of developing highly selective biopesticides [16]. Ultimately, the interplay between the physicochemical properties of sulfated fucans and their structural motifs governs their ability to interfere with membrane-bound protein interactions essential for pest survival, positioning these polysaccharides as compelling candidates for “blue chemistry” applications in sustainable agriculture. Having established the structural diversity of these metabolites, the following section explores how this chemical complexity translates into specific neurotoxic mechanisms in insect pests [24,25].

## 4. Molecular Mechanisms of Action in Agricultural Pests

The transition of bioactive metabolites into viable agrochemical agents requires a detailed understanding of their molecular mechanisms of action, particularly their ability to disrupt physiological homeostasis in target pests. Marine-derived compounds from Phaeophyceae, notably phlorotannins and sulfated polysaccharides, exert their bioactivity primarily through the selective inhibition of key enzyme systems involved in neuromodulation and metabolic detoxification [26,27].

### 4.1. Enzymatic Inhibition: Neuromodulation and Detoxification Pathways

Acetylcholinesterase (AChE) serves as the primary regulatory enzyme within the cholinergic synapses of agricultural pests, where it facilitates the rapid hydrolytic termination of the neurotransmitter acetylcholine (ACh) (Figure 2). Mechanistic inhibition of AChE activity by bioactive metabolites involves competitive or noncompetitive occupation of the enzyme’s active site—specifically targeting the catalytic triad or the peripheral anionic site—thereby obstructing the hydrolysis of ACh. This molecular blockade leads to the persistent accumulation of acetylcholine within the synaptic cleft, inducing chronic overstimulation of postsynaptic nicotinic and muscarinic receptors. The subsequent physiological manifestation is a state of uncontrolled neuromuscular hyperexcitation, resulting in paralysis and eventual lethality [28].

Molecular docking and in silico studies have investigated these interactions mechanistically (Figure 3), revealing key hydrogen bonds and hydrophobic contacts that stabilize inhibitor–AChE complexes. Such interactions have been documented in economically important pest species, such as *P. americana* and *S. oryzae*, underscoring the relevance of these metabolites as neurotoxic agents [28].

Beyond neuromodulation, the metabolic adaptability of insects and mites is largely governed by phase II detoxification enzymes, particularly glutathione S-transferases (GSTs). These enzymes catalyze the conjugation of reduced glutathione (GSH) to electrophilic xenobiotics, facilitating their conversion into water-soluble metabolites that can be readily excreted. GSTs also play a central role in mitigating oxidative stress by neutralizing reactive oxygen species (ROS) generated during metabolic challenge. Increased GST activity is a hallmark of insecticide resistance, with multiple isoforms—especially within the Delta and Sigma classes—being strongly associated with metabolic resistance in *P. americana* and other pest species. Notably, these isoforms have also been implicated as sources of allergenicity in humans [29].

Exposure to entomopathogenic fungi, such as *Beauveria bassiana*, further induces GST expression as part of a compensatory detoxification response [30]. Consequently, the targeted inhibition of GSTs represents a strategic approach to overcoming resistance. The coapplication of GST inhibitors alongside biopesticides has demonstrated considerable potential for synergistic effects, significantly enhancing overall toxicological efficacy [31].

### 4.2. Disruption of Midgut Homeostasis

The physiological integrity of the insect midgut is critically dependent on the peritrophic membrane (PM), which is a semipermeable chitin–protein matrix that regulates nutrient absorption while shielding the intestinal epithelium from mechanical damage and biochemical stress. Phlorotannins disrupt midgut homeostasis through their strong affinity for proteins, leading to the formation of insoluble tannin–protein complexes [32,33].

This interaction induces structural alterations in the PM, compromising its permeability and impairing the translocation of nutrients from the endoperitrophic space to the ectoperitrophic space. Simultaneously, digestive enzymes are sequestered through complexation with phlorotannins, resulting in reduced catalytic efficiency within the gut lumen. Collectively, these effects severely impair nutrient assimilation and metabolic efficiency, ultimately leading to developmental retardation and increased mortality in herbivorous insect pests [32,33].

### 4.3. Elicitation of Plant Defense Responses

In addition to their direct effects on pests, marine-derived fucans and sulfated polysaccharides from Phaeophyceae act as potent exogenous elicitors of plant innate immunity. These cell wall-derived macromolecules are recognized by plant pattern recognition receptors (PRRs), triggering a cascade of defense-related signaling events. The initial response is characterized by an oxidative burst, marked by the rapid accumulation of reactive oxygen species (ROS), such as hydrogen peroxide (H_2_O_2_) [34,35].

This early signaling event activates key enzymes, including phenylalanine ammonia-lyase (PAL) and lipoxygenase (LOX), which are central to the biosynthesis of salicylic acid (SA) and jasmonic acid (JA), respectively [34,35]. The subsequent systemic propagation of these hormonal signals promotes the expression of pathogenesis-related (PR) proteins, thereby establishing systemic acquired resistance (SAR) and induced systemic resistance (ISR) [36,37].

Such immune priming has proven effective against a broad spectrum of phytopathogens, including viruses such as Tobacco mosaic virus (TMV). Although SA–JA crosstalk is governed by complex regulatory networks and may involve antagonistic interactions, their coordinated activation is essential for providing a comprehensive defense against both biotrophic and necrotrophic stressors [36,37]. In this context, fucans function as molecular “primers” that enhance endogenous plant defense pathways, contributing to improved crop resilience. The diverse bioactivities of Phaeophyceae-derived metabolites and their molecular targets are summarized in Table 2.

### 4.4. Comparative Analysis with Commercial Biopesticides

To establish the potential of Phaeophyceae-derived metabolites as viable agrochemical leads, benchmarking their performance against mainstream commercially available biopesticides is essential. Table 3 provides a comprehensive comparison between phlorotannins/fucans and established biological agents, such as *Bacillus thuringiensis* (Bt), azadirachtin (Neem), and *Bacillus subtilis* [45,46].

While commercial agents such as Bt provide unparalleled potency against specific targets, Phaeophyceae-derived compounds offer a dual-action benefit by functioning as both direct biopesticides and plant biostimulants. This “blue chemistry” advantage provides a competitive edge in integrated pest management (IPM) systems; however, challenges regarding environmental persistence and cost-effective extraction remain the primary bottlenecks for technical optimization. While these molecular mechanisms are effective at the laboratory scale, their successful implementation in pest control strategies remains contingent upon field stability and formulation efficiency, as discussed in the subsequent section [48].

## 5. Advance Delivery Systems for Marine Leads

The transition of marine-derived bioactive compounds from laboratory-scale efficacy to practical agricultural applications is frequently constrained by the intrinsic chemical instability of these metabolites. Although crude extracts from Phaeophyceae often exhibit pronounced biological activity under controlled experimental conditions, their performance in open-field environments is typically compromised by the rapid degradation of their active constituents [49,50].

### 5.1. Challenges: Photodegradation and Bioavailability

One of the principal limitations associated with marine-derived polyphenols, particularly phlorotannins, is their pronounced sensitivity to environmental stressors. The high density of hydroxyl groups and extended conjugated systems of these compounds render them especially vulnerable to photodegradation upon exposure to ultraviolet (UV) radiation. Furthermore, oxidative stress and temperature fluctuations under field conditions can accelerate deleterious processes such as uncontrolled polymerization or molecular cleavage, resulting in a substantial loss of biopesticidal or elicitor activity [51].

Beyond chemical instability, the bioavailability and bioaccessibility of these metabolites are further restricted by their limited permeability across biological membranes and their propensity to form irreversible complexes with soil particles or plant surface components prior to reaching their physiological targets. Consequently, crude extracts often lack the persistence and consistency required for effective pest management, underscoring the necessity for advanced formulation strategies capable of preserving molecular integrity and enhancing delivery efficiency [51,52].

### 5.2. Nanoencapsulation Strategies

Nanoencapsulation has emerged as a powerful and versatile approach for overcoming these formulation challenges, providing an effective platform for improving the stability, UV protection, and controlled release of marine bioactive compounds. By entrapping sensitive metabolites within protective matrices, nanodelivery systems increase bioavailability while simultaneously reducing environmental losses and degradation [51,52].

Central to these strategies is the use of natural biopolymers that align with the principles of “blue chemistry”. Alginate, a polysaccharide extracted directly from brown algae, is widely employed owing to its excellent biocompatibility, low toxicity, and ability to undergo mild ionic gelation. Alginate-based micro- and nanoparticles offer a flexible structural framework that shields encapsulated bioactive compounds from premature degradation and enables sustained release profiles [53]. This approach is frequently complemented by chitosan, a cationic polymer derived from chitin, which is valued for its mucoadhesive properties and its ability to increase membrane permeability. The electrostatic assembly of alginate–chitosan polyelectrolyte complexes results in synergistic improvements in particle stability, encapsulation efficiency, and delivery performance, ensuring that bioactive compounds remain intact until they reach their intended target sites [54,55].

Furthermore, lipid-based and polymeric nanoparticles provide an effective barrier against intense UV radiation, functioning as physical and chemical shields that scatter or absorb incident light, thereby mitigating photodegradation. Studies employing polymeric nanoparticles have demonstrated that highly sensitive polyphenols, such as quercetin, retain their antioxidant activity and structural integrity for extended periods when encapsulated, resulting in markedly improved bioaccessibility in simulated biological systems [56]. Formulation techniques, including ionic gelation, spray drying, and emulsification, allow precise control over particle size, surface characteristics, and release kinetics, facilitating the sustained delivery of active compounds on plant surfaces or within the pest midgut [50,53]. Collectively, these advanced delivery systems provide a viable route for translating the unique metabolic profiles of Phaeophyceae into durable, high-performance agrochemical products [52,53,54,56].

## 6. Extracts Protocols and Green Chemistry

The laboratory-scale and industrial recovery of bioactive metabolites from Phaeophyceae has undergone a marked paradigm shift, moving away from resource-intensive conventional extraction methods toward approaches firmly grounded in the principles of green chemistry. Traditional techniques, including maceration and Soxhlet extraction, are increasingly regarded as suboptimal for marine-derived leads because of their reliance on large volumes of potentially hazardous organic solvents, extended extraction times, and the increased risk of thermal degradation of thermolabile compounds. In the case of phlorotannins and other sensitive polyphenols, such methods frequently promote oxidative reactions or irreversible structural modifications of the phenolic backbone, resulting in a substantial reduction in bioactivity and chemical stability [57].

In contrast, modern green extraction technologies—such as ultrasound-assisted extraction (UAE), microwave-assisted extraction (MAE), and supercritical fluid extraction (SFE)—increase efficiency, selectivity, and environmental sustainability. UAE exploits acoustic cavitation phenomena, whereby the formation and collapse of microbubbles generate localized pressure and temperature gradients that mechanically disrupt the algal cell wall. This process significantly improves mass transfer by facilitating solvent penetration, leading to increased extraction yields while minimizing processing time and avoiding excessive thermal stress [58,59].

Similarly, MAE employs microwave radiation to induce rapid and homogeneous heating through the dielectric rotation of polar molecules within the extraction matrix. This internal heating mechanism accelerates solvent diffusion and enhances extraction efficiency, enabling the rapid recovery of bioactive metabolites under tightly controlled conditions that preserve structural integrity [60]. Supercritical fluid extraction, particularly when supercritical CO_2_ is used, represents one of the most selective and environmentally benign extraction strategies currently available. By adjusting the pressure and temperature beyond the critical point, the solvent density and polarity can be finely tuned to target specific classes of metabolites. This technique operates at relatively low temperatures and yields solvent-free extracts, thereby protecting thermosensitive phlorotannins from degradation pathways, such as hydrolysis or uncontrolled polymerization, that are often associated with conventional solvent evaporation steps [61].

The choice of solvent system is equally critical for maintaining chemical integrity. While traditional organic solvents may compromise phenolic stability through oxidative mechanisms, greener alternatives—including aqueous ethanol, natural deep eutectic solvents (NADES), and supercritical CO_2_ supplemented with polar cosolvents—enable milder extraction conditions that preserve molecular conformation and antioxidant capacity [59,61]. Ultimately, the integration of these advanced extraction methods ensures that the recovery process is not only environmentally sustainable but also optimized for maximal retention of structural fidelity, thereby establishing a robust foundation for the development of high-performance marine-derived agrochemicals. To maximize the bioactivity identified in the previous sections, it is crucial to optimize these extraction methods; accordingly, the following section details the delivery systems designed to increase extract stability [62,63].

## 7. Critical Analysis and Future Perspectives

The successful transition of marine-derived bioactive compounds from laboratory-scale research to commercially viable agricultural products requires a rigorous and multidimensional critical assessment of the barriers that currently hinder market implementation. Despite the well-documented bioactivity of phlorotannins and fucoidans, several structural, regulatory, and technological challenges must be addressed before these marine leads can be effectively integrated into large-scale agricultural systems [47].

One of the most significant technical constraints lies in the standardization of raw marine extracts. In contrast to synthetic agrochemicals, which are produced under tightly controlled conditions and exhibit consistent chemical compositions, the metabolome of Phaeophyceae displays pronounced metabolic plasticity. The concentration, distribution, and structural profiles of secondary metabolites are strongly influenced by environmental variables such as sea surface temperature, light availability, salinity, nutrient gradients, and seasonal cycles. This inherent geographical and temporal variability complicates reproducibility in field trials and poses substantial challenges for industrial quality control and dosage calibration. Consequently, achieving the chemical uniformity required for regulatory approval and commercial scalability remains a critical bottleneck in the development of marine-derived biopesticides [47].

In parallel, the regulatory landscape represents a decisive factor in determining the commercial feasibility of these biobased solutions. Existing legislative frameworks, including the European Pesticide Regulation (EC) No. 1107/2009, frequently impose regulatory requirements on biopesticides that are comparable to those applied to conventional synthetic pesticides. Although regulatory initiatives have recently begun to recognize and promote “low-risk substances”, the registration process remains complex, costly, and time-intensive. The extensive data requirements for toxicological evaluation and environmental risk assessment can discourage industrial investment, particularly when the active principle consists of chemically complex extracts rather than a single, well-defined molecule [64].

Beyond regulatory and compositional challenges, the practical deployment of marine biopesticides is further constrained by limitations in terms of shelf life and environmental persistence. Compared with synthetic alternatives, natural compounds are often vulnerable to rapid biodegradation by soil microbiota and photoinactivation under solar radiation, which may necessitate more frequent applications [45]. Addressing these limitations will depend on the systematic validation of advanced formulation strategies—such as nanoencapsulation, controlled-release systems, and carrier-assisted delivery—through robust, multiseason field trials that accurately reflect agricultural conditions [65]. These efforts are essential for translating laboratory efficacy into consistent and reliable performance. Despite the potential of these optimized extracts, several industrialization bottlenecks remain; the following discussion addresses these challenges and proposes directions for future research.

### 7.1. Standardization and Quality Control

The transition to the industrial scale requires robust metabolomic profiling. The implementation of HPLC–DAD–MS/MS is essential for quantifying marker compounds, such as Dieckol or specific fucose-rich fractions [66]. Standardized detection protocols ensure that seasonal variations in Phaeophyceae populations do not compromise batch-to-batch efficacy [67]. Furthermore, large-scale cultivation in integrated multitrophic aquaculture (IMTA) systems offers a pathway to regulate metabolite uniformity and ensure sustainable biomass production [68].

### 7.2. Regulatory Frameworks and Registration

Commercial adoption of marine biopesticides faces significant regional hurdles:

- European Union: Under Regulation (EC) No. 1107/2009, substances must meet strict “low-risk” criteria, requiring comprehensive dossiers that prove a lack of endocrine disruption and minimal impact on nontarget organisms [64,69].
- United States: The EPA classifies these products as “biochemical pesticides”. Registration requires data on biocompatibility and environmental fate, categories where marine metabolites generally demonstrate favorable profiles [70].
- China: Recent regulatory shifts prioritize “green pesticides”, offering fast-track registration for biobased formulations that achieve field control efficacy exceeding 70% [71].

### 7.3. Shelf Life and Nanostabilization

Data indicate that free phlorotannins are highly susceptible to oxidation, potentially resulting in the loss of up to 50% of their biological activity within 30 days at room temperature [72]. However, the use of alginate-chitosan nanodelivery systems resulted in a 92% activity retention rate after 180 days of storage. This enhanced persistence is critical for achieving the stability required for commercial distribution and effective field application [73].

## 8. Conclusions

The metabolism of Phaeophyceae highlights a transformative opportunity for contemporary agriculture, offering a rich, diverse, and chemically sophisticated repertoire of secondary metabolites that reconciles effective pest management with environmental sustainability. The unique chemical architecture of phlorotannins and sulfated polysaccharides, including fucoidans, enables a dual mode of action distinct from conventional synthetic agrochemicals.

These marine metabolites directly affect agricultural pests through enzymatic inhibition and disruption of midgut homeostasis while simultaneously acting as biological elicitors that activate systemic defense responses in plants.

This multifunctional bioactivity is fully consistent with the principles of “blue chemistry,” which emphasize the responsible exploitation of renewable marine resources to address pressing global challenges, such as pesticide resistance and ecosystem degradation. By reducing the dependence on synthetic inputs, marine-derived biopesticides offer a pathway toward lowering the ecological footprint of crop protection strategies without compromising agronomic efficiency.

The advancement of these marine leads toward commercial implementation is strongly supported by the integration of modern technological platforms. Green extraction techniques, including supercritical fluid extraction and ultrasound-assisted methods, enable the recovery of bioactive fractions with high structural integrity, whereas innovative nanoencapsulation systems based on alginate and chitosan effectively mitigate issues related to photodegradation, environmental instability, and limited bioavailability. Nevertheless, persistent challenges associated with extract standardization and the complexity of international regulatory frameworks remain significant obstacles.

Ultimately, the successful integration of Phaeophyceae-derived metabolites into integrated pest management (IPM) programs will depend on coordinated advances in metabolic characterization, formulation science, and regulatory harmonization. By aligning these scientific and technological developments with sustainable production models, the agricultural sector can accelerate the transition toward a biobased economy that prioritizes ecological resilience while safeguarding global food security. The development of marine-derived agrochemicals therefore represents not merely a substitution of synthetic pesticides but also a fundamental shift toward a more holistic, resilient, and scientifically grounded paradigm of sustainable agriculture.

## Figures and Tables

**Figure 1:**
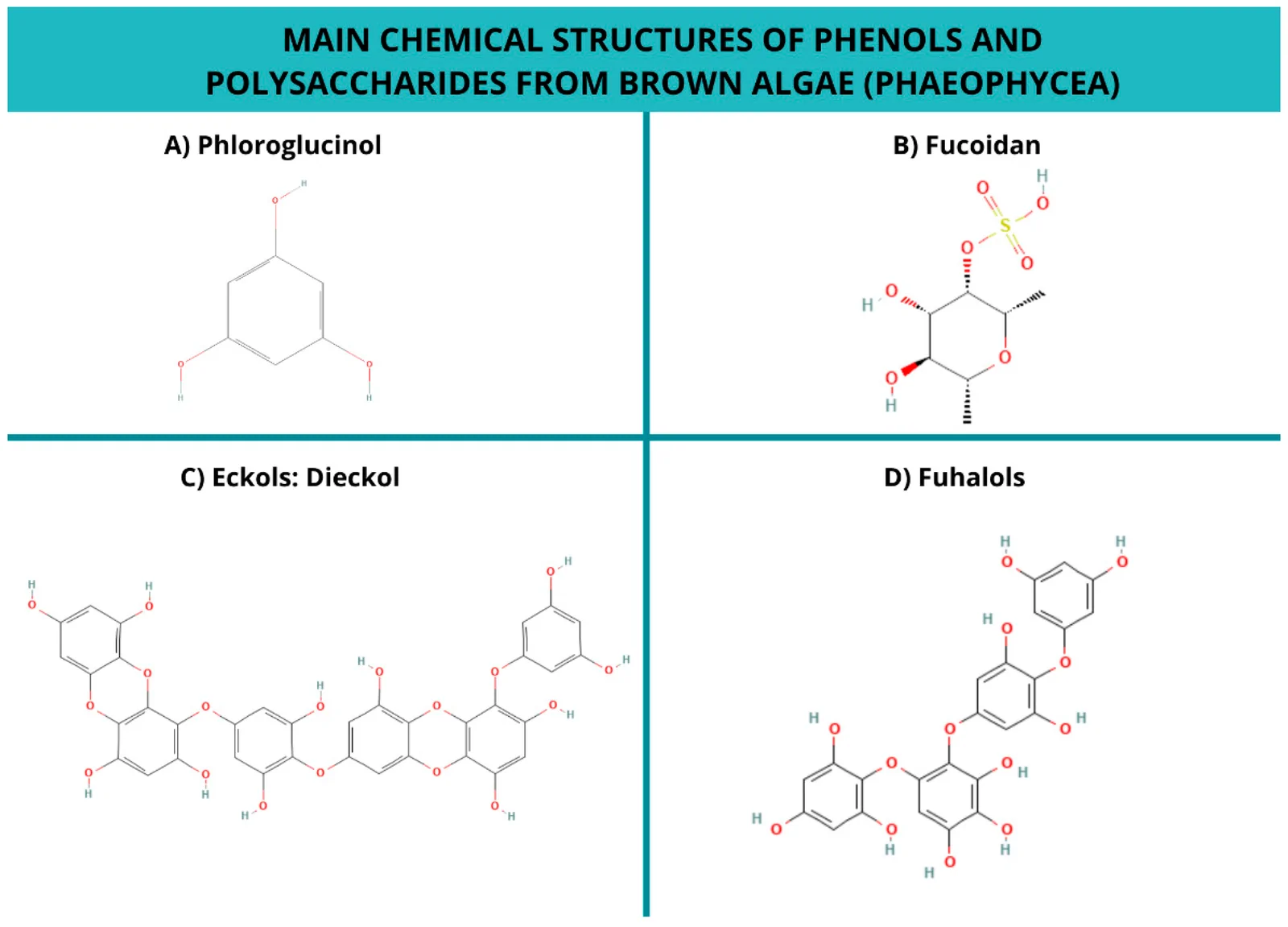
Structural diversity and biosynthetic pathways of phlorotannins in Phaeophyceae. (**A**) Chemical structure of the monomeric precursor, phloroglucinol (1,3,5-trihydroxybenzene). (**B**) Representation of the primary oxidative coupling modes: aryl–aryl (C-C) linkages characteristic of fucols and ether (C-O-C) linkages typical of phlorethols and fuhalols. (**C**) Structural hierarchy from simple oligomers to branched phlorethols, highlighting the increase in molecular complexity. (**D**) Model of a complex high-molecular-weight phlorotannin polymer, illustrating the dense network of hydroxyl groups and interunit linkages essential for cell-wall reinforcement and biological defense. Atom colors represent: carbon (grey), oxygen (red), and hydrogen (light blue). Created by the author on the Canva website.

**Figure 2:**
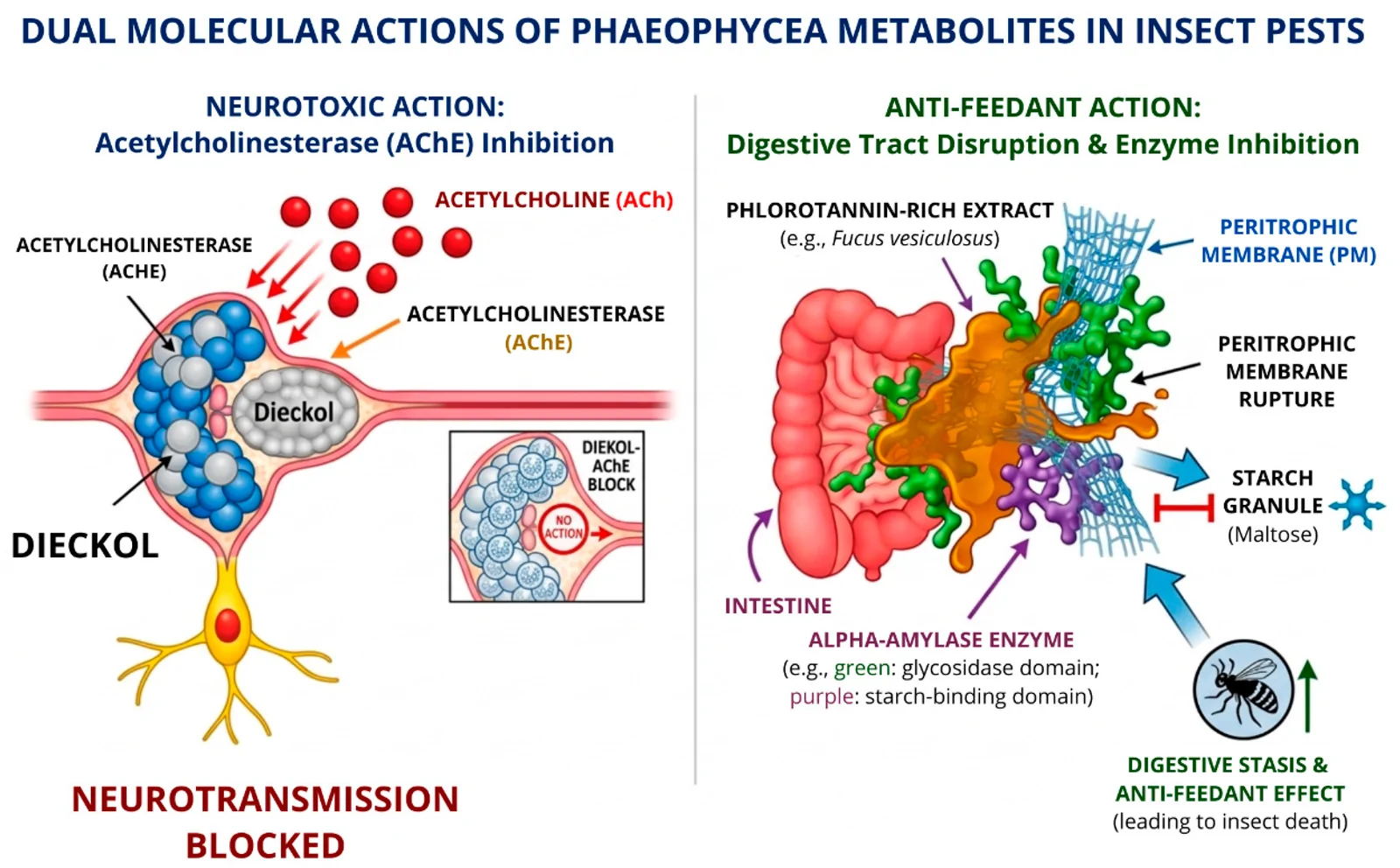
Dual molecular actions of Phaeophyceae metabolites in insect pests. The diagram details the neurotoxic and anti-feedant effects of brown algae extracts. On the left, Dieckol (represented in grey) acts on the synaptic cleft by inhibiting the enzyme acetylcholinesterase (AChE, blue/white spheres), which prevents the degradation of acetylcholine (Ach, red spheres) and blocks normal neurotransmission. On the right, the phlorotannin-rich extract (e.g., *Fucus vesiculosus*) causes peritrophic membrane (PM, blue lattice) rupture and inhibits the activity of the enzyme alpha-amylase (orange/purple structure), preventing starch digestion and resulting in digestive stasis and anti-food effects. Created by the author on the Canva website. The authors created these figures specifically for this review; no permission was needed.

**Figure 3:**
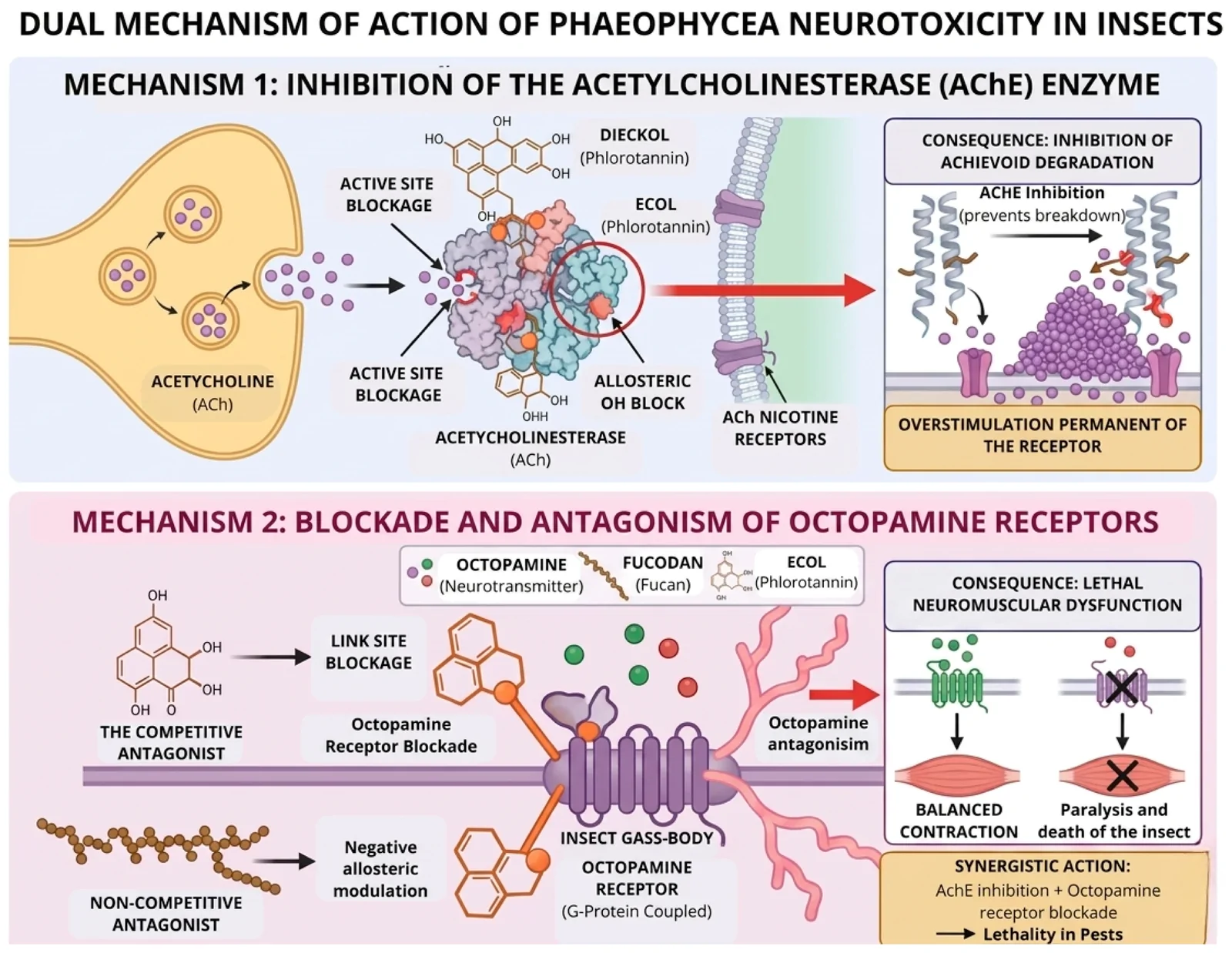
Dual mechanism of action of the neurotoxicity of phlorotannins and fucans in insect pests. The diagram illustrates the synergistic action of compounds extracted from Phaeophyceae (brown algae). In the upper part, the inhibition of the enzyme acetylcholinesterase (AChE) is observed, preventing the breakdown of the neurotransmitter acetylcholine (ACh). In the lower part, the blockade and antagonism of octopamine receptors is demonstrated, resulting in lethal neuromuscular dysfunction in the insect. Created by the author on the Canva website. The authors created these figures specifically for this review; no permission was needed.

**Table 1:** Comparative structural profile of phlorotannin groups identified in Phaeophyceae. The classification is based on the specific arrangement of monomeric phloroglucinol units and the chemical nature of their interunit linkages, which dictate their physicochemical properties and biological activities.

Class	Primary Linkage Type	Structural Characteristic	Representative Source Examples	References
**Fucols**	Aryl–Aryl (C-C)	Linear or branched polymers of phloroglucinol units.	*Fucus vesiculosus*, *Ascophyllum nodosum*	[17,18]
**Phlorethols**	Ether (C-O-C)	Units connected via oxygen atoms at varying positions.	*Cystoseira* species, *Sargassum*	[4,18]
**Fuhalols**	Ether (C-O-C)	Similar to phlorethols but with additional -OH groups.	*Bifurcaria bifurcata*, *Sargassum spinuligerum*	[17,19]
**Eckols**	Dibenzo-1,4-dioxin	Includes a tricyclic ring system with 1,4-dioxin moiety.	*Ecklonia cava*, *Eisenia bicyclis*	[4,15]
**Ishigeols**	Aryl–Aryl & Ether	Mixed linkages containing phloroglucinol and diphlorethol units.	*Ishige okamurae*	[15,20]

**Table 2:** Biological activity of bioactive metabolites and crude extracts from Phaeophyceae against significant agricultural pests and phytopathogens. The table correlates specific algal species and their primary metabolites with target organisms, elucidating the diverse molecular mechanisms—ranging from neurotoxicity and enzymatic inhibition to the elicitation of systemic plant defenses—that underpin their potential as sustainable agrochemical leads.

Compound/Extract	Source Species	Target Organism	Quantitative Indicators (lC_50_/LC_50_/Efficacy)	Extraction/Delivery Impact	References
Phlorotannins (Eckol)	*Ecklonia cava*	*Tetranychus urticae*	LC_50_ *: 1.25 mg/mL	15% increase in toxicity via Ultrasound-Assisted Extraction (UAE) *	[38]
Dieckol-rich extract	*Ecklonia stolonifera*	*Spodoptera litura*	LC_50_: 0.88 µg/larva	Enhanced stability when formulated in lipid-based nanocarriers	[39]
Sulfated Fucans	*Sargassum vulgare*	*Botrytis cinerea*	lC_50_ *: 0.45 mg/mL	75% field control efficacy when applied as a foliar spray	[40]
Fucoidan	*Fucus vesiculosus*	*Fusarium oxysporum*	MIC *: 1.20 mg/mL	Encapsulation in Chitosan-NPs * reduced the effective dose by 40%	[41]
Crude Phenolic Extract	*Sargassum muticum*	*Clavibacter michiganensis*	85% inhibition at 5.0 mg/mL	Microwave-Assisted Extraction (MAE)* preserved structural integrity	[42]
Laminarin	*Laminaria digitata*	*Plasmopara viticola*	75–90% reduction in disease severity	Systemic Acquired Resistance (SAR) elicitation observed in field trials	[43]
Phloroglucinol derivatives	*Cystoseira tamariscifolia*	*Aedes aegypti*	LC_50_: 15.4 ppm	High persistence in aquatic environments due to slow-release delivery	[44]

* Abbreviations: LC_50_ (median lethal concentration); lC_50_ (half-maximal inhibitory concentration); MIC (minimum inhibitory concentration); UAE (ultrasound-assisted extraction); MAE (microwave-assisted extraction); NPs (nanoparticles).

**Table 3:** Comparative analysis of Phaeophyceae-derived metabolites and mainstream commercial biopesticides.

Feature	Phaeophyceae Metabolites	*Bacillus thuringiensis* (Bt)	Azadirachtin (Neem)	*Bacillus subtilis*	References
Potency	High (comparable to botanicals)	Very High (highly targeted)	Moderate to High	Moderate (Protective)	[15,41]
Target Range	Broad (Insecticide/Fungicide)	Narrow (Lepidoptera)	Broad (IGR)	Broad (Antifungal)	[40,41,45]
Stability/Persistence	Low (needs nanoprotection)	Low (UV-sensitive)	Moderate	High (endospore-forming)	[41,45]
Environmental Impact	Positive (Biostimulant effect)	Neutral	Neutral	Neutral	[43,45,46]
Cost of Production	Moderate (Marine harvesting)	Low (Fermentation)	Moderate	Low (Fermentation)	[45,46,47]

## Data Availability

Data availability statements are available in this section.

## Abbreviations

The following abbreviations are used in this manuscript:

SAR	Structure–activity relationship
-OH	Hydroxyl
DP	Degrees of polymerization
AChE	Acetylcholinesterase
MIC	Minimum inhibitory concentration
CWDEs	Cell wall-degrading enzymes
ACh	Acetylcholine
GSTs	Glutathione S-transferases
GSH	Glutathione
ROS	Reactive oxygen species
PM	Peritrophic membrane
PRRs	Plant pattern recognition receptors
PAL	Phenylalanine ammonia-lyase
LOX	Lipoxygenase
SA	Salicylic acid
JA	Jasmonic acid
PR	Pathogenesis-related
ISR	Induced systemic resistance
TMV	Tobacco mosaic virus
UV	Ultraviolet
UAE	Ultrasound-assisted extraction
MAE	Microwave-assisted extraction
SFE	Supercritical fluid extraction
NADES	Natural deep eutectic solvents
EC	European pesticides regulation
IPM	Integrated pest management
IMTA	Integrated multitrophic aquaculture
EC	Under regulation
